# Decreased reaction time variability is associated with greater cardiovascular responses to acute stress

**DOI:** 10.1111/psyp.12617

**Published:** 2016-02-19

**Authors:** Andrew J. Wawrzyniak, Mark Hamer, Andrew Steptoe, Romano Endrighi

**Affiliations:** ^1^ Psychiatry and Behavioral SciencesUniversity of Miami Miller School of MedicineMiamiFloridaUSA; ^2^ National Centre for Sport & Exercise MedicineLoughborough UniversityLoughboroughUK; ^3^ Department of Epidemiology and Public HealthUniversity College LondonLondonUK; ^4^ Department of Medical & Clinical PsychologyUniformed Services University of the Health SciencesBethesdaMarylandUSA

**Keywords:** Reaction times, Cardiovascular, Acute stress, Reactivity and recovery

## Abstract

Cardiovascular (CV) responses to mental stress are prospectively associated with poor CV outcomes. The association between CV responses to mental stress and reaction times (RTs) in aging individuals may be important but warrants further investigation. The present study assessed RTs to examine associations with CV responses to mental stress in healthy, older individuals using robust regression techniques. Participants were 262 men and women (mean age = 63.3 ± 5.5 years) from the Whitehall II cohort who completed a RT task (Stroop) and underwent acute mental stress (mirror tracing) to elicit CV responses. Blood pressure, heart rate, and heart rate variability were measured at baseline, during acute stress, and through a 75‐min recovery. RT measures were generated from an ex‐Gaussian distribution that yielded three predictors: mu‐RT, sigma‐RT, and tau‐RT, the mean, standard deviation, and mean of the exponential component of the normal distribution, respectively. Decreased intraindividual RT variability was marginally associated with greater systolic (*B* = −.009, *SE* = .005, *p* = .09) and diastolic (*B* = −.004, *SE* = .002, *p* = .08) blood pressure reactivity. Decreased intraindividual RT variability was associated with impaired systolic blood pressure recovery (*B* = −.007, *SE* = .003, *p* = .03) and impaired vagal tone (*B* = −.0047, *SE* = .0024, *p* = .045). Study findings offer tentative support for an association between RTs and CV responses. Despite small effect sizes and associations not consistent across predictors, these data may point to a link between intrinsic neuronal plasticity and CV responses.

Much research has implicated increased or sustained cardiovascular (CV) responses to acute mental stress as detrimental to CV outcomes (Chida & Steptoe, 2010; Panaite, Salomon, Jin, & Rottenberg, 2015; Schwartz et al., 2003; Treiber et al., 2003). For example, greater blood pressure reactivity to, and recovery from, acute mental stress has been prospectively associated with the progression of atherosclerotic plaques, greater intima media thickness, and hypertension (Chida & Steptoe, 2010; Panaite et al., 2015). Mechanistically, individuals characterized by exaggerated CV responses to acute mental stress in the laboratory setting may respond in a similar fashion to natural stressors in everyday situations. This sustained hemodynamic activity may, over time, increase tonic blood pressure and lead to hypertension and related metabolic disorders. Additionally, other physiological processes including dysfunctional proinflammatory responses and sustained hypothalamic‐pituitary‐adrenal (HPA) axis reactivity have been associated with future development of CV disease risk factors (Brydon & Steptoe, 2005; Hamer, Endrighi, Venuraju, Lahiri, & Steptoe, 2012; Steptoe & Marmot, 2006).

In the psychophysiology field, reaction time (RT) measures are often obtained to index cognitive ability, but a potentially novel use of RTs may be as a proxy measure of neural efficiency (NE). This refers to the effectiveness with which the neural apparatus communicate and process information (Jensen, 2006). On a basic level, greater NE is represented by a smaller variability in RTs or a lower intraindividual mean (Neubauer & Fink, 2009) suggesting that a simple RT computation may not sufficiently capture the cognitive process involved. Regardless of the way one chooses to assess RT, research on RTs and CV outcomes is sparse. Recent epidemiological work (Hagger‐Johnson, Deary, Davies, Weiss, & Batty, 2014) observed that slower and more variable RT was associated with an increased risk of all‐cause and CV disease mortality in 5,134 adults from the NHANES III (National Health and Nutrition Examination Survey) study adjusting for age, sex, and ethnic minority status. Limited work has also examined the relationship between RTs or cognitive ability and a wide range of CV responses to acute mental stress that are implicated in cardiac risk. Ginty and colleagues (Ginty, Phillips, Der, Deary, & Carroll, 2011a) measured cognitive ability and simple RT at baseline in a large community sample of individuals 55 years and over, and blood pressure and heart rate reactivity to acute stress at 7‐year follow‐up. Results showed that low cognitive ability and slow RT were significant predictors of blunted heart rate stress reactivity after accounting for covariates including sociodemographics and medication use. In further analyses using a different age cohort (Ginty et al., 2011b), lower heart rate responses to acute stress were associated with slower RT at 5‐ and 12‐year follow‐up independent of covariates including baseline heart rate, socioeconomic position, and cohort type. Blood pressure responses to acute stress were not associated with RT in either study. In the Dutch Famine Birth Cohort Study (Ginty, Phillips, Roseboom, Carroll, & Derooij, 2012), it was observed that impaired cognitive ability was associated with a blunted CV response to acute stress. Therefore, slow RT and low cognitive ability seems to be associated with lower heart rate responses to acute stress. Crucially, according to the allostatic load framework (McEwen & Seeman, 1999), exaggerated autonomic responses to stress is regarded as maladaptive. However, some recent work is also exploring the notion that blunted or diminished reactivity may be a marker of heightened chronic stress resulting in disturbances of biological systems. For example, blunted CV and inflammatory stress responses were observed in otherwise healthy individuals with Type II diabetes (Steptoe et al., 2014). Furthermore, others have reported that blunted stress reactivity was associated with adverse health‐related outcomes including substance addiction, eating disorders, and depressive disorders (Phillips, Ginty, & Hughes, 2013).

To date, no study has examined the relationship between different RT measures and a wide range of CV responses to acute mental stress in a healthy, aging sample. In addition, CV recovery from acute stress and heart rate variability, which are important predictors of CV risk (Panaite et al., 2015; Villareal, Li, & Massumi, 2002) have not been examined. Few studies have examined RTs and CV reactivity and recovery as separate phenomena whereby the cognitive measures are independent of the acute stressor designed to elicit CV changes (Ginty et al., 2012). Such a study may help understand whether RTs predict a wide range of CV responses to stress independently of known covariates for reactivity and recovery.

Therefore, the present study examined the association between several RT measures modeled using an ex‐Gaussian distribution (Vaurio, Simmonds, & Mostofsky, 2009) and CV responses to mental stress in a healthy sample of participants drawn from the Whitehall II epidemiological cohort (Marmot et al., 1991). We hypothesized that RTs would be associated with hemodynamic and cardiac reactivity to, and recovery from, acute mental stress independently of a wide range of covariates.

## Method

### Participants and Design

A subsample of participants from the Whitehall II epidemiological cohort was recruited between 2006 and 2009 for a psychophysiologic study of acute stress responses and future CV disease risk factors. Exclusion criteria included a history or objective signs of coronary heart disease, a diagnosis or current treatment for hypertension, inflammatory diseases, cancer treatment in the past 5 years, or a current diagnosis or treatment for a mental health disorder. Up‐to‐date medical records were used to verify participants’ health characteristics to meet the inclusion criteria.

Participants gave informed consent to participate in the study, and ethical approval was obtained from the Joint University College London/University College London Hospital Research Ethics Committee. Participants were prohibited from using any antihistamine or anti‐inflammatory medications for 7 days prior to psychophysiological testing, and were rescheduled if they presented with colds or other sign of infection on their research appointment day. In addition, they were instructed to not consume caffeinated beverages or tea for at least 2 h prior to their visit, and to not partake in vigorous physical activity nor consume alcoholic beverages in the day prior to their appointment.

The current analytic sample consists of 262 participants with complete RT measures. The original study sample included 543 individuals with psychophysiologic stress data, but the RT study component was introduced part way through the study. Participants with RT data did not differ from the rest of the sample in regard to average age and body mass index (BMI), baseline blood pressure, heart rate and heart rate variability, distribution of gender, or employment grade (*p* value range = .16–.86). In addition, there were no between‐groups differences in blood pressure, heart rate, and heart rate variability responses to mental stress (*p* value range = .42–.86).

### Materials

Participants’ height and weight were measured by a research nurse according to a standardized protocol to determine participants’ BMI (kg/m^2^). The latest grade of employment was used as an index of socioeconomic status (SES; Steptoe & Marmot, 2002). Detailed RT data were obtained from a modified Stroop color‐naming task (Stroop, 1935), whereas CV responses were elicited using a mirror‐tracing task. Order of task presentation was counterbalanced whereby half of the participants completed the Stroop task followed by mirror‐tracing while the other half completed the tasks in the reverse order.

#### RT data assessment

RTs were obtained from participants’ performance on the Stroop color‐naming task (Heathcote, Popiel, & Mewhort, 1991), which was administered for 5 min. The task involved successive presentations of target color words at the top of a computer screen for 500 ms. The target words were printed in a discordant color (e.g., the word *green* printed in blue ink), and participants had to press a computer key that corresponded to the name of the target color word among a choice of four colors printed at the bottom of the screen (yellow, blue, red, and green). They were required to respond as fast and accurately as possible. The computer recorded each participant's RT between presentation of target words and the pressing of the key as well as whether or not the correct key was pressed. Since RT task duration was standardized across participants (5 min), different numbers of trials were presented. These raw RT data were used to compute three RT variables (see below). Participants were provided with standard written instructions and were allowed to practice for 1 min prior to task initiation. They were also instructed not to talk during the duration of the task.

#### Acute mental stress

Acute stress was elicited using a mirror‐tracing task (Campden Instruments Ltd.). Participants were instructed to trace around the marked contour of a star with an electronic stylus while looking at the star's reflection in a mirror. The apparatus beeped and recorded an error every time the participant deviated from the marked contour. Performance was determined by the number of times the drawing of the star was completed, as well as the number of errors made during the drawing. Participants were told that an average person completes the drawing five times in the 5 min allowed with a minimum number of mistakes. Standard written instructions were provided, and participants were allowed to practice the tasks for 1 min.

#### Stress‐induced cardiovascular reactivity and recovery assessment

Cardiovascular outcomes as indices of sympathetic activation to the heart (Kapuku et al., 1999) included systolic blood pressure (SBP), diastolic blood pressure (DBP), and heart rate (HR). Heart rate variability (HRV) expressed as the root mean square of successive N‐N differences (r‐MSSD, ms) was used as a measure of parasympathetic influence on the heart. SBP and DBP were monitored continuously from the finger during the entire testing session using an appropriately calibrated Finometer (TNO Biomedical Instrumentation, Amsterdam, The Netherlands), which employs the vascular unloading technique (Imholz, Wieling, van Montfrans, & Wesseling, 1998). Beatscope software was used for data reduction and to compute SBP and DBP variables.

HR and HRV were measured continuously using an ActiHeart monitoring device (Cambridge Neurotechnology, UK) attached to the participant's chest with electrocardiogram (ECG) electrodes. The ActiHeart records both HR and movement; validity has been reported during running and resting conditions (Brage, Brage, Franks, Ekelund, & Wareham, 2005). The raw data were reduced and analyzed using the HRV Analysis Software (Biomedical Signal Analysis Group, University of Kuopio, Finland).

### Procedure

Psychophysiological testing was carried out at either 9:30 am or 1:00 pm. A research nurse ensured that participants were not suffering from a cold or a viral infection and that the pretesting instructions had been followed. Anthropometric measures were obtained according to standard protocol, and participants were escorted to a stress laboratory where they sat in a padded recliner for the entire duration of the testing protocol. After instrumentation, participants rested quietly for 30 min. CV assessment of the last 5 min of this rest period were averaged to provide baseline values.

**Figure 1:**
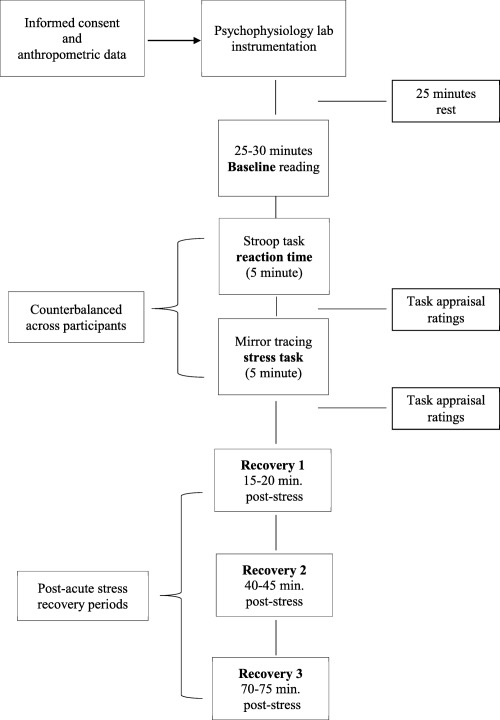
Graphic overview of study procedure.

A research assistant trained in psychophysiology subsequently administered the RT task and the mirror‐tracing task while CV data were continuously assessed. Self‐report ratings of task difficulty and task involvement were obtained immediately after each task. Participants were then required to rest quietly for 75 min during a stress recovery period while CV data were continuously assessed. Three time periods that were equally spaced between each other (15–20, 40–45, and 70–75 min poststress) were assessed to provide CV stress recovery values as per previous studies in our lab (Steptoe & Marmot, 2006, Steptoe et al., 2014), which adequately captures both short‐ and long‐term changes in recovery across multiple autonomic measures. During recovery, participants remained seated on the reclining armchair and were allowed to read nature magazines or watch nature DVDs (Figure 1).

### Statistical Analysis

#### Cardiovascular data

Reactivity to stress was computed by subtracting baseline values from the value obtained during the mirror‐tracing task so that higher scores reflect greater reactivity. Recovery from stress was computed by subtracting baseline values from the values obtained during each of the three time points into the recovery period so that greater scores indicate impaired recovery (slower return to baseline). HRV data were log‐transformed prior to analyses other than for the robust regressions. Distributions of change scores were visually screened for outliers, but no observation needed correction. The effect of acute stress on these CV outcomes was examined using repeated measures analysis of variance (ANOVA) with Greenhouse‐Geisser correction for degrees of freedom where appropriate.

#### RT data reduction

Only RTs for correct trials were used in the analyses (Silvia, Jones, Kelly, & Zibaie, 2011). Due to increased cognitive demands when errors are processed in the brain (Coles, Scheffers, & Holroyd, 2001; Falkenstein, Hoormann, Christ, & Hohnsbein, 2000; Koehn, Dickinson, & Goodman, 2008), RT slows during the second of two sequential incorrect trials (Hajcak & Simons, 2008). Therefore, incorrect trial times were excluded from the analysis. All RTs faster than 200 ms were considered as “anticipatory” errors and where thus removed. Additionally, RTs greater than four standard deviations above the individual mean were considered as outliers (Schmiedek, Oberauer, Wilhelm, Suss, & Wittman, 2007). Only eight participants required removal of one RT that was greater than four standard deviations, and a single case required the removal of two RTs. After removing these cases, all RTs were within four standard deviations of each participant's mean.

#### RT data modeling

Ex‐Gaussian distributions have been previously used to characterize RT data (Hervey et al. 2006; Leth‐Steensen, Elbaz, & Douglas, 2000; Vaurio et al., 2009). The ex‐Gaussian distribution of RTs can be described as the normal, or Gaussian, distribution, plus an independent, exponentially distributed variable. The mean and variance of the normal distribution along with the exponential component together form the ex‐Gaussian distribution (Heathcote et al., 1991; Leth‐Steensen et al., 2000). This distribution is comprised of mu (mu‐RT—a central tendency measure similar to the normal distribution's mean), sigma (sigma‐RT—a value for the normal distribution's variation), and tau (tau‐RT—the mean of the exponential component of the distribution) (Hervey et al., 2006).

In the ex‐Gaussian distribution, mu‐RT and sigma‐RT represent the mean and standard deviation, respectively, of the response times. Tau‐RT reflects the intraindividual variability whereby greater values indicate longer but infrequent response times. Examining RT data using the ex‐Gaussian method avoids the possibility of more variable RTs being considered as outliers or noise, and prevents the need to trim or log‐transform the data in attempts to fit a normal distribution (Hervey et al., 2006). Ex‐Gaussian distribution was calculated for all RTs using a maximum likelihood fitting system to generate mu‐RT, sigma‐RT, and tau‐RT variables for each participant. These RTs were then used as predictor variables of stress reactivity and stress recovery.

#### Covariates

Participants’ sociodemographics and physiological characteristics including age, sex, BMI, and grade of employment (Steptoe, Willemsen, Kunz‐Ebrecht, & Owen, 2003) known to have a direct impact on CV stress responses were used as covariates. In addition, number of correct Stroop trials (a marker of cognitive ability), stress task perceived difficulty (Sherwood, Davis, Dolan, & Light, 1992), and involvement (Silvia et al., 2011) were treated as covariates because it may influence stress responses. Participants rated task difficulty and involvement on a 7‐point Likert‐type scale but, due to the bimodal distribution of responses, we dichotomized it into a high/low binomial variable using a median split. Finally, the baseline value of the appropriate CV outcome was included in the models.

#### Robust regression

To examine the association between the ex‐Gaussian distribution of RTs for each participant and their CV responses to acute stress, we used robust regression. The highly robust and efficient SMDM regression estimator that provides a high breakdown point and 95% asymptotic efficiency for normal errors (Koller & Stahel, 2011) was used. This method has the advantage of assigning less weight to observations with large regression residuals, allowing the inclusion of all available data points. Notably, standardized betas are not provided in robust regressions in that the standardization assumes that all data points are equally weighted. However, *R* squared (*R* ^2^) was calculated using the unweighted and unscaled values of the predictors and outcomes (Street, Carroll, & Ruppert, 1988).

Descriptive statistics are presented as means (±*SD*) or percentage as appropriate. Analyses of associations between RT variables and CV stress responses are presented as *B* (±*SE*) and *R* ^2^ along with *p* values. Results were considered to be statistically significant at the ≤ .05 level, and were considered a trend at the < .1 level. Analyses were performed using R version 3.2.2 (R Core Team, 2015) and the R package ‘robustbase’ (Rousseeuw et al., 2013).

## Results

### Participant Characteristics

Participant sociodemographic characteristics and baseline CV values are presented in Table 1. Data were obtained from 262 participants (mean age = 63.3 ± 5.5 years; 61.1% female). Average BMI indicated that the sample was only slightly overweight (mean = 25.7 ± 4.0 kg/m^2^). Overall, participants were normotensive but only slightly above the optimal blood pressure cutoffs (mean SBP = 126.9 ± 16.1 mmHg; mean DBP = 74.6 ± 10.3 mmHg).

**Table 1:** Subject Characteristics

Variable	Mean ± *SD* or *n* (%)
Sex	
Male	102 (38.9)
Female	160 (61.1)
Age (yrs)	63.3 ± 5.5
BMI (kg/m^2^)	25.70 ± 4.0
Marital status	
Married	156 (59.5)
Not married	105 (40.1)
Current smoker	13 (5.0)
Ex‐smoker	72 (27.5)
Employment grade	
Higher	75 (28.6)
Intermediate	109 (41.6)
Lower	78 (29.8)
Baseline systolic blood pressure (mmHg)	126.9 ± 16.1
Baseline diastolic blood pressure (mmHg)	74.6 ± 10.3
Baseline heart rate (bpm)	67.2 ± 8.8
Baseline heart rate variability (ms)	23.7 ± 13.5

*n* = 262.

*Note*. Data are shown as mean ± *SD*. BMI = body mass index.

### Acute Mental Stress Effect

Acute stress elicited the expected, robust changes in CV activity (summarized in Table 2). Repeated measures ANOVA showed a significant main effect of time for SBP: *F*(1.99,500.33) = 316.54, *p* < .001; DBP: *F*(2.02,505.65) = 258.86, *p* < .001; and HR: *F*(1.48,340.55) = 489.65, *p* < .001. These three variables were lowest at baseline and highest during stress; neither SBP nor DBP returned to baseline values even at 75 min post stress. HR was significantly lower than baseline at 45 and 75 min after stress. There was also a main effect of time for HRV: *F*(2.24,456.18) = 68.21, *p* < .001. This measure significantly decreased in response to acute stress and then increased during the three recovery points.

**Table 2:** Summary of Average Cardiovascular Values at Baseline, During Stress, and During Recovery Periods

Variable	Baseline	Acute stress	Recovery 1 (+20 min)	Recovery 2 (+45 min)	Recovery 3 (+75 min)
SBP (mmHg)	126.88 ± 16.1	158.72 ± 23.5	137.87 ± 18.8	136.69 ± 18.7	138.00 ± 18.5
DBP (mmHg)	74.61 ± 10.3	89.57 ± 13.1	80.93 ± 11.4	80.38 ± 11.4	81.33 ± 11.2
HR (bpm)	67.15 ± 8.8	76.21 ± 10.3	66.43 ± 8.4	65.34 ± 8.6	65.69 ± 8.4
HRV (ms)	23.65 ± 13.5	18.22 ± 9.9	25.62 ± 13.0	26.43 ± 14.8	26.17 ± 14.4

*n* = 262.

*Note*. Data are shown as means ± *SD*. SBP = systolic blood pressure; DBP = diastolic blood pressure; HR = heart rate; HRV = heart rate variability.

### Stroop Task RT Performance

Mean percent (± *SD*) of correct trials was 63.64% (± .22). The mean (± *SD*) RT for the correct trials was 2,344.21 (± 39.86) ms.

### RT and Cardiovascular Stress Reactivity

A summary of the regression models for the RT predictors (mu‐RT, sigma‐RT, tau‐RT) and stress‐induced CV response outcomes is provided in Table 3.

**Table 3:** Summary of Robust Regressions Analyses Between Reaction Time (RT) Predictors and Cardiovascular Response Outcomes

		Stress reactivity		Stress Recovery 1 (+20 min)		Stress Recovery 2 (+45 min)		Stress Recovery 3 (+75 min)	
Outcome	RT predictor	*B* ± *SE*	*R* ^2^	*B* ± *SE*	*R* ^2^	*B* ± *SE*	*R* ^2^	*B* ± *SE*	*R* ^2^
SBP	mu‐RT	−.0026 ± .002	.05	−.002 ± .001	.05	−.003 ± .001**	.05	−.0027 ± .001**	.06
	sigma‐RT	−.0075 ± .006	.05	−.0046 ± .004	.04	−.00255 ± .004	.04	−.00253 ± .004	.06
	tau‐RT	−.009 ± .005**	.05	−.007 ± .003*	.06	−.006 ± .004	.04	−.006 ± .003**	.06
DBP	mu‐RT	−.00013 ± .001	.08	−.0003 ± .001	.06	−.0009 ± .001	.05	−.0001 ± .0007	.07
	sigma‐RT	−.00001 ± .002	.08	−.0008 ± .002	.06	−.00119 ± .002	.05	−.00030 ± .002	.07
	tau‐RT	−.0004 ± .002**	.10	−.0023 ± .001	.07	−.00192 ± .002	.05	−.00159 ± .002	.06
HR	mu‐RT	.0018 ± .001*	.13	.0007 ± .001**	.15	.0004 ± .0004	.18	.0009 ± .0005*	.20
	sigma‐RT	.00026 ± .002	.11	.0010 ± .001	.13	.00096 ± .001	.18	.0019 ± .001	.20
	tau‐RT	−.0030 ± .002	.11	−.0005 ± .001	.13	.0001 ± .000	.18	.00056 ± .001	.18
HRV	mu‐RT	−.0018 ± .001*	.72	−.0003 ± .001	.23	−.0021 ± .001*	.12	−.0015 ± .001**	.15
	sigma‐RT	−.0032 ± .002	.71	.00028 ± .002	.23	−.0028 ± .003	.11	−.0019 ± .002	.14
	tau‐RT	−.0038 ± .002**	.71	−.0003 ± .002	.24	−.0047 ± .002*	.12	−.0029 ± .002	.14

*n* = 262.

*Note*. Data are shown as mean ± *SE* and *R* ^2^ derived from robust regression. Mu‐RT is a measure of central tendency; sigma‐RT is a measure of variation of the normal distribution; tau‐RT is a measure of the mean of exponential component of distribution. Stress reactivity is a difference score between stress task and baseline values; stress recoveries are the difference scores between each poststress recovery value and baseline. Regression coefficients are adjusted for the baseline (prestress) value of the cardiovascular outcome, age, sex, BMI, employment grade, correct Stroop trials (cognitive ability), and self‐report stress task difficulty and involvement rating. SBP = systolic blood pressure; DBP = diastolic blood pressure; HR = heart rate; HRV = heart rate variability.

*p ≤ .05. **p ≤ .09.

Mu‐RT (ex‐Gaussian mean RT component) was not associated with SBP (*B* = −.0026, *SE* = .002, *p* = .24) or DBP (*B* = −.00013, *SE* = .001*, p* = .88) stress reactivity after controlling for study covariates. However, there was an association with HR (*B* = .0018, *SE* = .001, *p* = .03; model *R* ^2^ = .13, *p* = .029) indicating that slower RT was an independent predictor of greater stress‐induced HR increases; baseline HR and task difficulty rating were also significant covariates (*p* < .01). Additionally, changes in HRV were associated with slower mu‐RT (*B* = −.0018, *SE* = .001, *p* = .05, model *R* ^2^ = .72, *p* = .05) with baseline HRV, employment grade, and stress task appraisal as significant covariates (*p* < .05).

Sigma‐RT (ex‐Gaussian variance RT component) was not significantly associated with SBP (*B* = −.0075, *SE* = .006, *p* = .21), DBP (*B* = −.0001, *SE* = .002, *p* = 0.97), HR (*B* = .0026, *SE* = .0022, *p* = .23), or HRV (*B* = −.0032, *SE* = .0024, *p* = .19) stress reactivity after adjustment for covariates.

**Figure 2:**
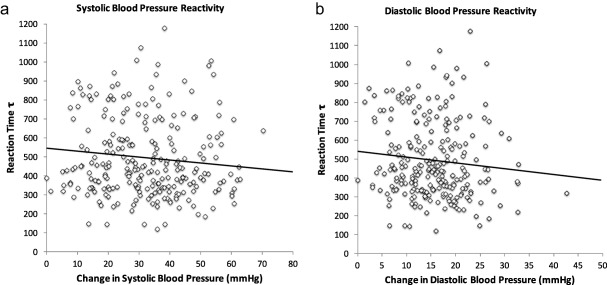
a: Scatter plot of the association between tau‐RT and systolic BP reactivity to acute mental stress (*n* = 262). b: Scatter plot of the association between tau‐RT and diastolic BP reactivity to acute mental stress (*n* = 262). Reaction time tau is expressed in milliseconds and represents the intraindividual variability in reaction times. Change in systolic and diastolic BP is the difference between baseline and acute stress values so that greater scores reflect higher stress‐induced reactivity. Individuals with lower intraindividual variability in reaction time tended to show marginally greater systolic BP (*p* = .09) and diastolic BP (*p* = .08) stress reactivity. The association is fully adjusted for age, sex, BMI, employment grade (SES), stress task perceived difficulty and involvement, correct Stroop RT trials (cognitive ability), and baseline BP.

Tau‐RT (ex‐Gaussian intraindividual variability RT component) was only marginally, inversely associated with SBP reactivity (*B* = −.009, *SE* = .005, *p* = .09; model *R* ^2^ = .05, *p* = .07; Figure 2a), tentatively suggesting that lower intraindividual variability in RT predicts greater stress‐induced increases in SBP, with BMI being a significant factor (*p* < .05). A similar pattern of marginally significant association was observed with DBP reactivity (*B* = −.004, *SE* = .002, *p* = .08; model *R* ^2^ = .10, *p* = .07; Figure 2b). HR reactivity was not significantly associated with tau‐RT (*B* = −.003, *SE* = .002, *p* = 0.15) and neither was HRV reactivity (*B* = −.0038, *SE* = .002, *p* = .10; model *R* ^2^ = .71, *p* = .09). However, baseline HRV, employment grade, and stress task involvement were significant covariates in the final model (*p* < .05).

### RT and Cardiovascular Stress Recovery

Mu‐RT was only marginally associated with SBP stress recovery at 45 min (*B* = −.003, *SE* = .001, *p* = .08; model *R* ^2^ = .05, *p* = .08) and 75 min (*B* = −.0027, *SE* = .001, *p* = .06; model *R* ^2^ = .06, *p* = .06), indicating that faster RT marginally predicted impaired poststress recovery. However, no association was evident 15 min poststress (*B* = −.002, *SE* = .0001, *p* = .15). There was no association with DBP stress recovery at 15 (*B* = −.0003, *SE* = .001, *p* = .63), 45 (*B* = −.0009, *SE* = .0007, *p* = .20), or 75 min poststress (*B* = −.001, *SE* = .0007, *p* = .19).

The association between mu‐RT and HR stress recovery only approached significance level at 15 min poststress (*B* = .0007, *SE* = .001, *p* = .09; model *R* ^2^ = .15, *p* = .09), but it became not significant at 45 min (*B* = .0004, *SE* = .0004, *p* = .35). However, at 75 min poststress, this association was significant (*B* = .0009, *SE* = .0005, *p* = .05; model *R* ^2^ = .20, *p* = .05) suggesting that slower RT predicted impaired HR recovery at the end of the stress period adjusting for study covariates (baseline HR and age were also significant factors, *p* < .01). Although there was no association between mu‐RT and HRV recovery at 15 min (*B* = −.003, *SE* = .001, *p* = .69), a significant association emerged at 45 min poststress (*B* = −.0021, *SE* = .00093, *p* = .02; model *R* ^2^ = .125), which then trended toward nonsignificance by the end of the stress protocol (*B* = −.0015, *SE* = .001, *p* = .088; model *R* ^2^ = .15, *p* = .07). Baseline HRV was also a significant factor (*p* < .001).

There were no significant associations between sigma‐RT and SBP, DBP, HR, or HRV stress recovery at any time point into the recovery period (*p* range = .126–.878).

**Figure 3:**
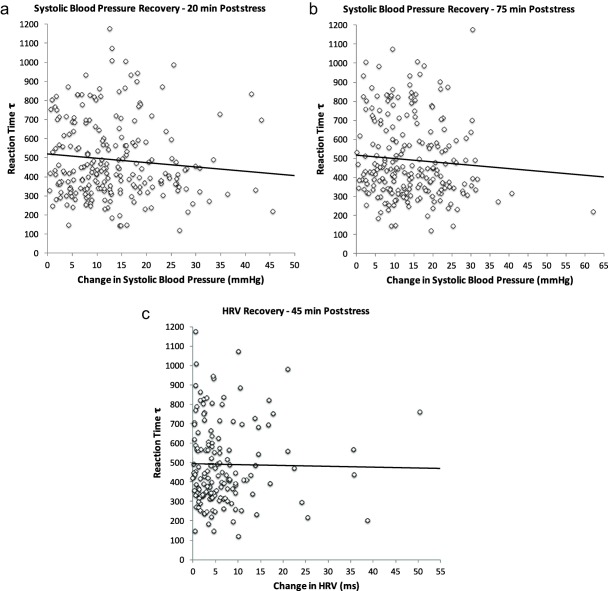
a: Scatter plot of the association between tau‐RT and systolic BP stress recovery at 20 min after acute stress (*n* = 262). b: Scatter plot of the association between tau‐RT and systolic BP stress recovery at 75 min after acute stress (*n* = 262). c: Scatter plot of the association between tau‐RT and heart rate variability stress recovery at 45 min after acute stress (*n* = 262). Reaction time tau is expressed in milliseconds and represents the intraindividual variability in reaction times. Change in SBP is the difference between the stress recovery time point and the baseline value so that greater scores indicate impaired recovery (delayed return toward baseline) from acute stress. Change in heart rate variability is the difference between the baseline and the 45‐min poststress value so that lower values reflect impaired recovery from acute stress. Individuals with lower intraindividual variability in reaction times showed impaired vascular and vagal recovery from acute stress. These associations are fully adjusted for age, sex, BMI, employment grade (SES), stress task perceived difficulty and involvement, correct Stroop RT trials (cognitive ability), and baseline BP and HRV.

Tau‐RT was associated with impaired SBP stress recovery at 15 min poststress (*B* = −.007, *SE* = .003, *p* = .03; model *R* ^2^ = .06, *p* = .03), indicating that lower intraindividual variability in RT predicted impaired SBP stress recovery independent of study covariates (Figure 3a). There was no significant association between tau‐RT and SBP stress recovery at 45 min (*B* = −.006, *SE* = .004, *p* = .13), but there was a marginally significant association at 75 min poststress (*B* = −.006, *SE* = .003, *p* = .087; model *R* ^2^ = .06, *p* = .08) (Figure 3b). Baseline SBP and correct Stroop trial were also significant factors (*p* < .04).

Tau‐RT was neither associated with DBP stress recovery at any time point into the recovery period (*p* range = .12–.38) nor with HR stress recovery (*p* range = .60–.99).

Finally, tau‐RT was associated with HRV stress recovery at 45 min after stress (*B* = −.0047, *SE* = .0024, *p* = .045, model *R* ^2^ = .12), indicating that lower intraindividual variability independently predicted impaired HRV stress recovery (Figure 3c). Baseline HRV was again a significant factor (*p* < .001).

## Discussion

The present study examined associations between three RT measures assessed with the Stroop task and modeled through an ex‐Gaussian distribution, and CV responses to acute mental stress in a healthy, older sample. We hypothesized that the three RT variables (mu‐RT, sigma‐RT, tau‐RT) would be associated with CV responses to acute stress. However, given the inconsistent findings in the literature, we did not predict the direction of these associations.

The results revealed a complex pattern of associations between the RT measures and stress‐induced responses that was not always consistent across predictors and was mostly modest in magnitude. The most consistent finding observed in these analyses was that sigma‐RT, a measure of variation in RT in a normal distribution, was not significantly associated with any of the CV reactivity and recovery outcomes at any time point (*p*s ≥ .12). In contrast, mu‐RT, a measure of average response time similar to the mean of a normal distribution, was associated with heart rate and heart rate variability stress reactivity, which is mostly consistent with previous work (Ginty et al., 2011a, 2011b, 2012). This indicated that individuals with slower RT had greater heart rate and (lower) heart rate variability responses to acute stress after adjustment for several covariates including age, sex, BMI, number of correct trials as a proxy of cognitive ability, subjective ratings of task engagement and difficulty, and employment grade. Since we also adjusted for the baseline (prestress) value of these cardiac markers, this result is not attributable to differences in resting cardiac autonomic activity in individuals with slower RT.

Furthermore, mu‐RT was a significant, independent predictor of impaired heart rate and heart rate variability recovery from stress at some, but not all, time points. This suggests that two important cardiac parameters failed to return to baseline values by the end of the stress protocol in individuals with slower RT responses. This finding was underscored by the marginally significant association between mu‐RT and impaired systolic BP recovery at 75 min (*p* = .06), although this association was not replicated with diastolic BP. This finding is novel and adds to the literature on RTs and stress responses by showing that individuals with slower RT had sustained cardiac and vascular activation (impaired recovery) following mental stress.

Tau‐RT, which can be characterized as the values describing both the mean and the standard deviation of the exponential component (greater values reflect longer but infrequent response time), was only weakly associated with both systolic and diastolic BP reactivity (Figure 2a,b). In contrast, no association was observed with heart rate and heart rate variability. However, this RT measure showed some interesting and novel associations with CV recovery. Firstly, there was an inverse association with impaired recovery in blood pressure at 15 and, partially, at 75 min poststress (Figure 3a,b), but not at 45 min. In addition, tau‐RT was associated with impaired vagal control at 45 min (Figure 3c). These findings suggest that individuals characterized by impaired or slower intraindividual variability in RTs show sustained vagal and vascular activation following mild mental stress. Again, our results add to the literature by showing associations with stress recovery parameters, although these relationships appeared to be weak and not always consistent across time points.

As suggested in the introduction, the concept of neuronal efficiency may be used as a framework to interpret our results. Neuronal efficiency is an element of intrinsic plasticity, or nonsynaptic factors, that directly impacts the probability that a neuron will fire an action potential and bears implication for health outcomes including addiction (Kourrich, Calu, & Bonci, 2015; Zhang & Linden, 2003). In the field of psychophysiology, the role of neuronal efficiency has been overlooked as a potential mechanistic facet of the central nervous system response to mental stress. Low variability in neuronal firing (indexed by a smaller tau‐RT) can be seen phenotypically as being associated with higher reactivity to a stressor (seen with blood pressure and heart rate variability), and then with a better return to baseline during recovery (systolic BP). Intrinsic plasticity may account for previous studies’ findings on RT such as in work associating lower cognitive ability and slower RT with blunted heart rate stress reactivity (Ginty et al., 2011a, 2011b) and associations between poorer cognitive ability and lower CV reactions to acute stress (Ginty et al., 2011a, 2011b). Further support for intrinsic neuronal plasticity as a factor in CV reactivity has been reported in fMRI studies that have observed associations between blunted reactivity and neural hypoactivation (Ginty, Gianaros, Derbyshire, Phillips, & Carroll, 2013).

### Strengths and Limitations

The present study has both limitations and strengths. This study's findings are bolstered by the fact that participants were carefully selected on the basis of being free of any objective sign of chronic diseases. As such, participants in the study may represent an unusually healthy sample of the population that may potentially limit the generalizability of the findings to a wider population. Although we controlled for a number of covariates and accurately implemented a reliable stress testing procedure, the cross‐sectional nature of the study cannot rule out the possibility that other unmeasured factors might have contributed to the findings. In addition, reverse causality is a possibility in that heightened reactivity could influence RT. Although this is plausible, further work would require an experimental manipulation of reaction times in order to determine the causal relationship between RTs and CV responses to mental stress. Importantly, the effect sizes observed in the study were modest and not always consistent across predictors, and it may be difficult to interpret them before further replication is achieved. Another limitation may be that our analyses were based on a subsample of participants with available RT data, and therefore statistical power to detect small associations might have been an issue. However, there were no significant differences between participants with and without RT data on several factors including CV responses to stress. Furthermore, we used time rather than frequency domain measures of heart rate variability, and we may have had different results if spectral analysis had been performed. Additionally, we did not measure breathing patterns; respiratory rate can impact heart rate variability, although parasympathetic modulation of heart rate not related to respiratory rate have been used to index stress responses (Houtveen, Rietveld, & de Geus, 2002).

Strengths of the study include the use of a mental stress protocol that included an adequate stress recovery period with continuous CV assessment. This protocol allowed us to investigate associations with stress recovery as well as reactivity that has seldom been examined in previous work (Ginty et al., 2011, 2012), but that is increasingly recognized as an important risk factor for CV morbidity and mortality (Panaite et al., 2015).

In summary, we have offered some preliminary evidence that RT measures are associated with CV responses to stress. Specifically, not only do average measures of RT relate to CV responses, but the variability in RTs, which can also be thought of as a proxy measure for neuronal efficiency, may be predictive of CV reactivity to, and recovery from, an acute stressor. Future studies, potentially using large, younger samples, are needed that implement noninvasive measures to record RTs and examine their variability in order to support the hypothesis that neuronal efficiency is related to CV stress responses.

The clinical implication would be that, if this hypothesis is supported, it may potentially provide insight into alternative means of measuring CV dysfunction. Since persistent and exaggerated CV responses to acute stressors are detrimental to long‐term health and can foster disease progression, determining reactivity and recovery to an acute stressor can be clinically informative. However, preclinical CV measurements to assess this dysfunction are typically cost prohibitive. Therefore, measuring neuronal efficiency through RTs may serve as an early, low‐effort, indirect measurement of CV reactivity.
